# Discrepancies in glycemic metrics derived from different continuous glucose monitoring systems in adult patients with type 1 diabetes mellitus

**DOI:** 10.1111/1753-0407.13296

**Published:** 2022-07-21

**Authors:** Yongwen Zhou, Xiaodong Mai, Hongrong Deng, Daizhi Yang, Mao Zheng, Bin Huang, Linlin Xu, Jianping Weng, Wen Xu, Jinhua Yan

**Affiliations:** ^1^ Department of Endocrinology and Metabolism, Guangdong Provincial Key Laboratory of Diabetology The Third Affiliated Hospital of Sun Yat‐sen University Guangzhou China; ^2^ Department of Endocrinology, The First Affiliated Hospital of USTC, Division of Life Sciences and Medicine University of Science and Technology of China Hefei China

**Keywords:** diabetes mellitus, type 1, glucose, glycemic control, 1型糖尿病, 血糖, 血糖控制

## Abstract

**Background:**

Continuous glucose monitoring systems have been widely used but discrepancies among various brands of devices are rarely discussed. This study aimed to explore differences in glycemic metrics between FreeStyle Libre (FSL) and iPro2 among adults with type 1 diabetes mellitus (T1DM).

**Methods:**

Participants with T1DM and glycosylated hemoglobin of 7%–10% were included and wore FSL and iPro2 for 2 weeks simultaneously. Datasets collected on the insertion and detachment day, and those with insufficient quantity (<90%) were excluded. Agreements of measurement accuracy and glycemic metrics were evaluated.

**Results:**

A total of 40 498 paired data were included. Compared with the values from FSL, significantly higher median value was observed in iPro2 (147.6 [106.2, 192.6] vs. 144.0 [100.8, 192.6] mg/dl, *p* < 0.001) and the largest discordance was observed in hypoglycemic range (median absolute relative difference with iPro2 as reference value: 25.8% [10.8%, 42.1%]). Furthermore, significant differences in glycemic metrics between iPro2 and FSL were also observed in time in range (TIR) 70–180 mg/dl (TIR, 62.8 ± 12.4% vs. 58.8 ± 12.3%, *p* = 0.004), time spent below 70 mg/dl (4.4 [1.8, 10.9]% vs. 7.2 [5.4, 13.3]%, *p* < 0.001), time spent below 54 mg/dl (0.9 [0.3, 4.0]% vs. 2.6 [1.3, 5.6]%, *p* = 0.011), and coefficient of variation (CV, 38.7 ± 8.5% vs. 40.9 ± 9.3%, *p* = 0.017).

**Conclusions:**

During 14 days of use, FSL and iPro2 provided different estimations on TIR, CV, and hypoglycemia‐related parameters, which needs to be considered when making clinical decisions and clinical trial designs.

## Graphical abstract

**Highlights**

- The accordance between the paired dataset between FSL and iPro2 was acceptable but poorer in hypoglycemic range.
- Significant differences for glycemic metrics between iPro2 and FSL were observed in time in range 70–180 mg/dL, coefficient of variation, and hypoglycemia‐related parameters (including time below range 54 mg/dL and 70mg/dL).
- The study reminded us when using the CGM for clinical decisions and clinical trials, it is necessary to consider the model heterogeneity.

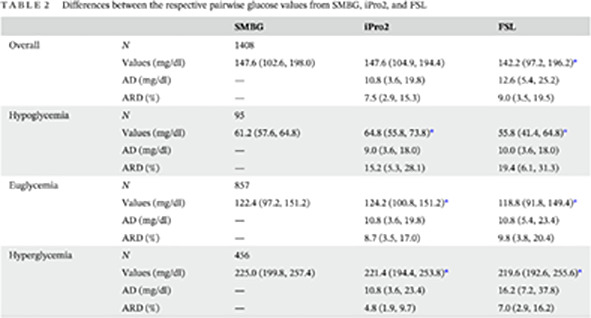

## 摘要

**背景:**

连续血糖监测系统已被广泛使用，但不同品牌的设备之间的差异很少被讨论。本研究旨在探讨成人1型糖尿病(T1DM)患者中Freestyle Libre(FSL)和iPro2在血糖指标上的差异。

**方法:**

选择T1DM和糖化血红蛋白(HbA1c)为7%~10%的受试者，同时使用FSL和iPRO 2两周。排除了在植入和取出当天收集的数据集，以及质量不够高(<90%)的数据集。对测量准确度和血糖指标的一致性进行评估。

**结果:**

共纳入40498对数据。与FSL相比，iPro2的中位数显著升高(147.6[106.2,192.6]vs 144.0[100.8,192.6]mg/dL，P<0.001)，其中不一致性最高的是低血糖范围(以iPro2为参考值的绝对差值中位数:25.8% [10.8%,42.1%])。此外，iPro2和FSL在70‐180 mg/dL达标时间(TIR)内的血糖指标也有显著差异(TIR，62.8±12.4vs.58.8±12.3%，P=0.004)，在70 mg/dL以下的时间(4.4 [1.8, 10.9]% vs.7.2[5.4，13.3]%，P<0.001])，在54 mg/dL以下的时间(0.9[0.3，4.0]% vs.2.6[1.3，5.6]%, p=0.011)，变异系数(CV, 38.7±8.5% vs.40.9±9.3%，P=0.017)。

**结论:**

在使用的14天中，FSL和iPro2对TIR、CV和低血糖相关参数提供了不同的估计，在临床决策和临床试验设计时需要考虑这些因素。

## 1 INTRODUCTION

Looking through the history of diabetes management, diabetes technologies have progressed significantly and played a vital role for patients and caregivers in modern society. The continuous glucose monitoring (CGM) systems, recording the interstitial fluid glucose every 5–15 mins and thus generating the glucose profiles, have been accepted gradually and widely used worldwide. As reported in the Type 1 Diabetes Exchange Registry study, the number of CGM users rose from 7% in 2010–2012 to 30% in 2016–2018 in the United States.^1^ Since 2017, the CGM has been recommended to use in conjunction with insulin therapy among patients with type 1 diabetes mellitus (T1DM).^2^

With the large amounts of CGM data collected, the glycemic metrics generated from CGM data such as time in range (TIR) 70–180 mg/dl, time below range (TBR) <70 mg/dl, time above range (TAR) >180 mg/dl, and glycemic variability are of great importance for the assessment of glycemic control and complications management.^3^ Therefore, accuracy and reliability in CGM devices had a great priority in enabling authentic benefits and safety. Nowadays, there are various brands of CGM devices on the market, but their features are heterogeneous, including the time intervals of data collection and the calibration methods. Besides, published evidence had also shown that heterogeneity of accuracy in different kinds of devices is large with the mean absolute relative difference (ARD) of 9.9% in professional iPro2, 8.5%–13.0% in real‐time CGM systems, and 10.7%–11.4% in FreeStyle Libre (FSL), especially in the hypoglycemic range.^4^, ^5^, ^6^, ^7^, ^8^ With the potential brand‐to‐brand differences in glucose levels, whether the recommended device‐derived metrics were also affected was unknown.

Previous studies have assessed the CGM metrics derived from different brands of CGM devices,^9^, ^10^, ^11^ but few had been conducted between the professional CGM and intermittently scanned CGM systems,^12^ both of which are devices frequently used in the clinical outpatient visits. Furthermore, evidence had indicated that the longer duration of glucose data analyzed could provide better estimations of glycemic metrics. Most of these previous studies used the 7‐day CGM data instead of the currently recommended 14‐day duration.^13^ Therefore, in this study, we used 14 days of glucose data collected from two parallel worn CGM systems (FSL and iPro2) in the registered clinical trial and evaluated their accordance in measurement accuracy and glycemic metrics.

## 2 MATERIALS AND METHODS

### 2.1 Subjects

**FIGURE 1:**
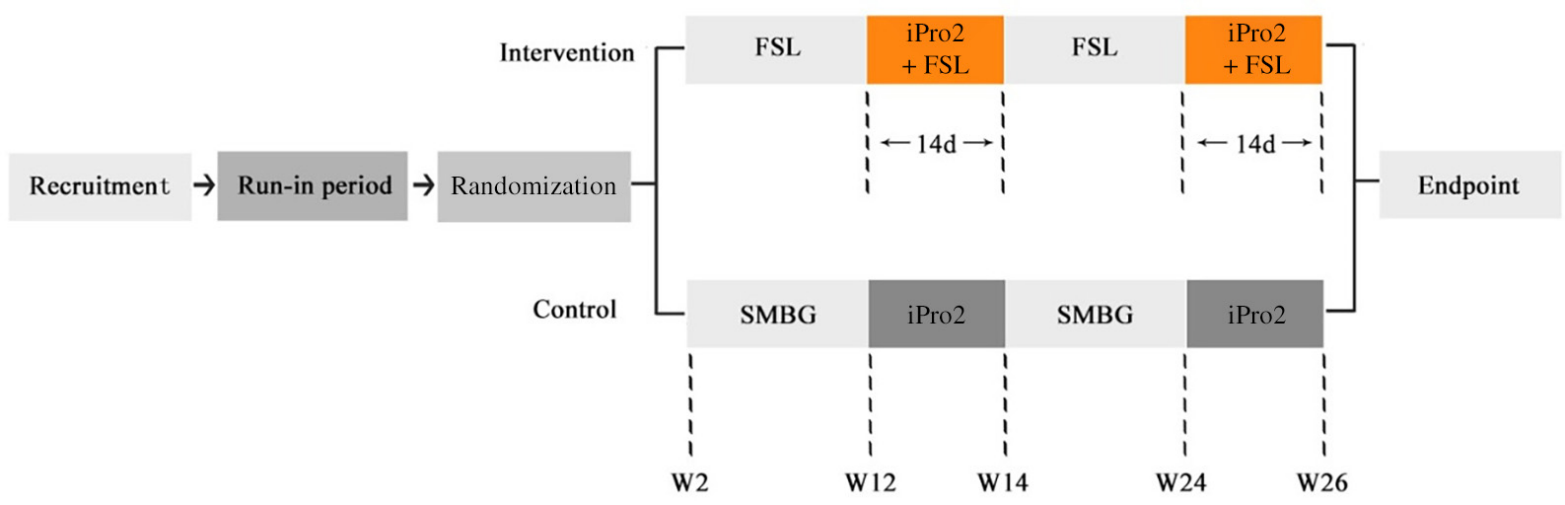
Flow chart of the trial with 12–14 and 24–26 weeks data sets extracted and analyzed in this study. The data analyzed in this study were collected from the intervention group during the follow‐up period at week 12–14 and week 24–26. FSL, FreeStyle Libre; SMBG, self‐monitoring of blood glucose.

All data used in the article were obtained from an open‐label, randomized clinical trial (NCT03522870) that was described in the previous article,^14^ and the glucose data collected from the intervention group during the follow‐up period at weeks 12–14 and week 24–26 were analyzed in the present study (Figure 1). Adult patients (≥18 years) with T1DM, glycosylated hemoglobin (HbA1c) 7%–10%, and multiple daily injections or continuous subcutaneous insulin infusion therapy were recruited. The main exclusion criteria were the use of any CGM system 3 months before study entry, known allergy to medical‐grade adhesives or CGM devices and its affiliated components, and being pregnant or planning pregnancy. This trial was approved by the Ethics Committee of the Third Affiliated Hospital of Sun Yat‐sen University (approval number: [2017] 2–5) and was accordant with the Declaration of Helsinki. All subjects were given oral explanations and written informed consent.

### 2.2 Experimental procedure and data collection

During the follow‐up period, all patients wore the FSL and iPro2 simultaneously for the consecutive 2 weeks (equal to one FSL sensor and two Sof‐sensors). The sensors were implanted into the subcutaneous tissues of the upper arms on the same or opposite side, without preference. During the 2 weeks, patients still intermittently scanned the FSL sensor but were additionally required to test the finger‐stick blood glucose ≥3 times per day for calibrations of the simultaneously wearing iPro2 using the blood glucose meter (Bayer®; Bayer Consumer Care AG). All device‐related adverse events described previously were recorded.^15^ Both sensors were detached after 14 days and glucose data were downloaded via the respective software. Demographic and anthropometric data, including body mass index, were collected on the day of insertion via the case report forms.

### 2.3 Data analysis

#### 2.3.1 Data processing

Datasets with sufficient available quantity (≥90%) were included in the analysis. Data collected on the day of sensor insertion and detachment were removed because of the less stable performance reported previously.^16^ The accordance and correlations between the two devices were analyzed based on the paired data sets. The value from the FSL was matched with the closest iPro2 value from within the setting lag time of 2.5 min. The pairs with the sensor value exceeding the highest threshold in iPro2 (400 mg/dl) were removed. The value of self‐monitoring of blood glucose (SMBG) was matched with the closest paired data with the lag time <7.5 min.

#### 2.3.2 Performance analysis

Performance analysis of devices was assessed via the following statistical methods with paired data: (a) absolute difference (AD) and ARD. The AD and ARD against the SMBG value were determined as follows: AD (mg/dl) = |CGM − SMBG|, ARD (%) = (|CGM − SMBG|)/ SMBG × 100. To directly compare FSL and iPro2, the discordances between FSL and iPro2 were also calculated as follows: AD (mg/dl) = |FSL − iPro2|, ARD (%) = (|FSL − iPro2|)/ iPro2 × 100. (b) Parkes error grid. Parkes error grid was divided into five zones based on estimated clinical risks, with glucose data falling into Zone A and B and thus representing little or no effect on clinical practice.^17^

#### 2.3.3 Calculation of clinical metrics

Glycemic metrics were calculated via Glyculator 2.0 software using unpaired data.^18^ The day and night periods were set as 06:00–24:00 and 00:00–06:00. According to the recommendations of International Consensus,^19^ the following metrics were analyzed: (a) euglycemia: TIR 70–180 mg/dl; (b) hypoglycemia: TBR (defined as range <70 or <54 mg/dl) and low blood glucose index (LBGI)^20^; (c) hyperglycemia: TAR (defined as range >180 or >250 mg/dl) and high blood glucose index (HBGI)^20^; and (d) glycemic variability: SD, coefficient of variation (CV), mean amplitude of glucose excursion, and mean of the daily differences.^21^ The LBGI and HBGI are usually used to quantify the risk of hypo‐ and hyperglycemia and are shown to be predictive of meaningful glycemic events.^20^, ^22^

### 2.4 Statistics

Data were presented as means ± SD with normal distributions and medians (interquartile range) with nonnormal variables. The correlation between the two systems was tested via R^2^ calculated in linear regression models. Pairwise comparisons of medians between the paired glucose readings derived from SMBG, FSL, and iPro2 were performed using related samples Wilcoxon signed‐rank test. For the comparisons of glycemic metrics, paired‐samples *t* test and related‐samples Wilcoxon signed‐rank test were used for normal distributions parameters and nonnormally distributed parameters, respectively. All statistical analyses used SPSS version 23 software (SPSS Inc., Chicago, IL, USA), and *p* < 0.05 was considered statistically significant. The Parkes error grid was done using Matlab 2014 software.

## 3 RESULTS

### 3.1 Participants and CGM performance

From May 2018 to November 2020, a total of 44 datasets were collected from 27 patients with 26 datasets collected in week 12–14 and 18 datasets collected in week 24–26. As presented in Table 1, the median age was 29.8 (21.2, 36.2) years, with a median duration of diabetes 10.0 (6.2, 13.9) years and a mean HbA1c level of 7.5 ± 0.7%. The mean duration of available datasets analyzed in this study was 9.6 ± 2.1 days.

**TABLE 1:** Characteristics of patients included in the analysis

Characteristics	
Age (years)	29.8 (21.2, 36.2)
Male/female	10/34
Duration (years)	10.0 (6.2, 13.9)
BMI (kg/m^2^)	21.2 (20.0, 23.1)
HbA1c (%)	7.5 ± 0.7
Hemoglobin (g/L)	133.2 ± 12.5
MDI (*n*/%)	29 (65.9)
CSII (*n*/%)	15 (34.1)
Daily insulin dosage (IU/kg/day)	0.7 (0.6, 0.8)

Abbreviations: BMI, body mass index; CSII, continuous subcutaneous insulin infusion; IU, international units; MDI, multiple daily injection.

### 3.2 Agreement of the paired values from SMBG, iPro2, and FSL

Taking the SMBG value as the reference, there were a total of 1408 SMBG‐iPro2‐FSL paired data sets retracted. The overall correlation between the two devices and SMBG values was satisfactory (iPro2, R^2^ = 0.89; FSL, R^2^ = 0.81). Compared with the overall median of SMBG values (147.6 [102.6, 198.0] mg/dl), the median of FSL values was significantly lower (142.2 [97.2, 196.2] mg/dl, *p* < 0.001), whereas the difference between the SMBG and iPro2 values was not significant. The median ARD for FSL was significantly higher than that for iPro2 (9.0% [3.5, 19.5] vs. 7.5% [2.9, 15.3]; *p* < 0.001). When stratifying glucose values based on the different SMBG ranges, the highest median ARD was observed in the hypoglycemia group (iPro2, 15.2% [5.3, 28.1]; FSL, 19.4% [6.1, 31.3]; Table 2).

**TABLE 2:** Differences between the respective pairwise glucose values from SMBG, iPro2, and FSL

		SMBG	iPro2	FSL
Overall	*N*	1408		
	Values (mg/dl)	147.6 (102.6, 198.0)	147.6 (104.9, 194.4)	142.2 (97.2, 196.2)*
	AD (mg/dl)	—	10.8 (3.6, 19.8)	12.6 (5.4, 25.2)
	ARD (%)	—	7.5 (2.9, 15.3)	9.0 (3.5, 19.5)
Hypoglycemia	*N*	95		
	Values (mg/dl)	61.2 (57.6, 64.8)	64.8 (55.8, 73.8)*	55.8 (41.4, 64.8)*
	AD (mg/dl)	—	9.0 (3.6, 18.0)	10.0 (3.6, 18.0)
	ARD (%)	—	15.2 (5.3, 28.1)	19.4 (6.1, 31.3)
Euglycemia	*N*	857		
	Values (mg/dl)	122.4 (97.2, 151.2)	124.2 (100.8, 151.2)*	118.8 (91.8, 149.4)*
	AD (mg/dl)	—	10.8 (3.6, 19.8)	10.8 (5.4, 23.4)
	ARD (%)	—	8.7 (3.5, 17.0)	9.8 (3.8, 20.4)
Hyperglycemia	*N*	456		
	Values (mg/dl)	225.0 (199.8, 257.4)	221.4 (194.4, 253.8)*	219.6 (192.6, 255.6)*
	AD (mg/dl)	—	10.8 (3.6, 23.4)	16.2 (7.2, 37.8)
	ARD (%)	—	4.8 (1.9, 9.7)	7.0 (2.9, 16.2)

Abbreviations: AD, absolute difference; ARD, absolute relative difference; FSL, FreeStyle Libre; SMBG, self‐monitoring of blood glucose.

* Means *p* < 0.05, compared with the values from the device and from SMBG.

### 3.3 Discordances in glycemic values between iPro2 and FSL

Among the total of 40 498 paired datasets between iPro2 and FSL, the correlation between the two devices was less satisfactory but still acceptable (R^2^ = 0.74, *p* < 0.001). The overall median AD and ARD between the two devices were 18.0 (9.0, 34.2) mg/dl and 13.1 (6.0, 24.3)%, respectively (Table 3). Compared with the values from FSL, the significantly higher median glucose value was observed in iPro2 (147.6 [106.2, 192.6] vs. 144.0 [100.8, 192.6] mg/dl; *p* < 0.001). When stratifying glucose ranges based on the iPro2 values, the largest discordance was observed in the hypoglycemia group (median ARD with iPro2 as reference value: 25.8 [10.8, 42.1]%) whereas the values were more accordant in the euglycemia and hyperglycemia groups (median ARD with iPro2 as reference value: 14.0 [6.5, 25.6]% and 10.3 [4.7, 18.9]%, respectively). Parkes error grid analysis was also performed in the paired data sets (Figure S1). A total of 98.1% paired FSL‐iPro2 values fell within Zones A and B and there were 1.6% and 0.3% of paired values falling within Zones C and D.

**TABLE 3:** Median AD and ARD between paired readings derived from two devices

		iPro2	FSL
Overall	*N*	40 498	
	Values (mg/dl)	147.6 (106.2, 192.6)*	144.0 (100.8, 192.6)
	AD (mg/dl)	18.0 (9.0, 34.2)	
	ARD (%)	13.1 (6.0, 24.3)	
Hypoglycemia	*N*	2654	
	Values (mg/dl)	57.6 (48.6, 64.8)*	59.4 (43.2, 77.4)
	AD (mg/dl)	14.4 (5.4, 25.2)	
	ARD (%)	25.8 (10.8, 42.1)	
Euglycemia	*N*	25 531	
	Values (mg/dl)	127.8 (102.6, 153.0)*	122.4 (93.6, 154.8)
	AD (mg/dl)	18.0 (7.2, 30.6)	
	ARD (%)	14.0 (6.5, 25.6)	
Hyperglycemia	*N*	12 313	
	Values (mg/dl)	219.6 (196.2, 253.8)*	214.2 (185.4, 248.4)
	AD (mg/dl)	23.4 (10.8, 43.2)	
	ARD (%)	10.3 (4.7, 18.9)	

Abbreviations: AD, absolute difference; ARD, absolute relative difference; FSL, FreeStyle Libre.

* Means *p* < 0.05, compared with the values from the two devices.

### 3.4 Discrepancies of the CGM metrics between the two devices

For the unpaired glucose readings collected from FSL and iPro2, there were a total number of 44 399 and 133 366 sensor readings used for the calculation of glycemic metrics respectively. Overall, significant differences were observed in TIR, hypoglycemic metrics, and CV. Compared with the metrics in iPro2, TIR was significantly lower in FSL group (58.8 ± 12.3% vs. 62.8 ± 12.4%, difference: −4.0 [95% confidence interval (CI) = −6.7 to −1.4]%; *p* = 0.004). The hypoglycemic metrics, including TBR < 54 mg/dl, TBR < 70 mg/dl and LBGI, were all significantly higher in FSL group, with the differences of 2.1 (95% CI = −0.5 to 3.7)%, 3.7 (95% CI = −1.6 to 5.8)% and 0.9 (95% CI = −0.3 to 1.5)% respectively (TBR < 54 mg/dl: 2.6 [1.3, 5.6]% vs. 0.9 [0.3, 4.0]%; TBR < 70 mg/dl: 7.2 [5.4, 13.3]% vs. 4.4 [1.8, 10.9]%; and LBGI: 1.7 [1.4, 3.1] vs. 1.1 [0.5, 2.8]; *p* < 0.05, all). In addition, significantly higher CV in FSL group was also observed (40.9 ± 9.3 vs. 38.7 ± 8.5%, *p* = 0.017). Differences in other glycemic metrics were not observed between the two devices.

Data in daytime and nighttime were also analyzed respectively. As shown in Table 4, differences in hypoglycemic metrics in the respective periods were both significant with the higher ones in the FSL group and the differences were extremely high in the nighttime with 3.8% in TBR < 70 mg/dl and 3.2% in TBR < 54 mg/dl_._ Besides, in the daytime, TIR was also lower in the FSL group (58.5 ± 11.7 vs. 63.0 ± 11.9%, *p* = 0.002) and CV was significantly higher, whereas in the nighttime both of these metrics were similar (*p* > 0.05). Of note, in the nighttime, beyond the differences in hypoglycemia, the mean glucose values and the TAR > 250 mg/dl in the FSL group were significantly lower (*p* <0.05 for both).

**TABLE 4:** Glycemic metrics between FSL and iPro2 system in the monitoring period, daytime and nighttime

	All				Daytime (06:00–24:00)				Nighttime (00:00–06:00)			
	FSL	iPro2	Difference (95% CI)	*p*	FSL	iPro2	Difference (95% CI)	*p*	FSL	iPro2	Difference (95% CI)	*p*
Mean (mg/dl)	150.7 ± 25.5	155.3 ± 23.1	−4.6 (−9.8, 0.6)	0.080	154.6 ± 24.3	158.4 ± 21.3	−3.8 (−9.1, 1.5)	0.155	138.8 ± 34.9	145.9 ± 34.1	−7.1 (−13.0, −1.2)	0.020
Median (mg/dl)	144.3 ± 29.1	148.1 ± 23.9	−3.8 (−9.1, 1.5)	0.150	148.5 ± 26.8	150.9 ± 22.5	−2.4 (−7.7, 2.9)	0.370	133.8 ± 39.4	138.1 ± 35.6	−4.3 (−10.7, 2.1)	0.185
eA1c (%)	6.9 ± 0.9	7.0 ± 0.8	−0.2 (−0.3, 0.0)	0.080	7.0 ± 0.9	7.1 ± 0.7	−0.1 (−0.3, 0.1)	0.155	6.5 ± 1.2	6.7 ± 1.2	−0.2 (−0.5, 0.0)	0.020
*Hypoglycemia*												
TBR (<70 mg/dl) (%)	7.2 (5.4, 13.3)	4.4 (1.8, 10.9)	3.7 (1.6, 5.8)	0.001	7.1 (3.6, 12.7)	3.2 (1.3, 9.4)	3.6 (1.6, 5.6)	0.001	11.3 (3.5, 19.0)	6.0 (2.0, 16.7)	3.8 (0.6, 7.0)	0.007
TBR (<54 mg/dl) (%)	2.6 (1.3, 5.6)	0.9 (0.3, 4.0)	2.1 (0.5, 3.7)	0.011	2.0 (0.5, 5.1)	0.7 (0.2, 3.3)	1.7 (0.3, 3.2)	0.034	5.0 (0.4, 9.4)	1.4 (0, 5.4)	3.2 (0.5, 5.9)	0.011
LBGI	1.7 (1.4, 3.1)	1.1 (0.5, 2.8)	0.9 (0.3, 1.5)	0.003	1.7 (0.9, 2.9)	0.8 (0.4, 2.4)	0.8 (0.3, 1.4)	0.003	3.1 (1.0, 4.5)	1.4 (0.6, 3.6)	1.2 (0.2, 2.2)	0.007
*Euglycemia*												
TIR (70–180 mg/dl) (%)	58.8 ± 12.3	62.8 ± 12.4	−4.0 (−6.7, −1.4)	0.004	58.5 ± 11.7	63.0 ± 11.9	−4.5 (−7.2, −1.8)	0.002	59.9 ± 17.9	62.2 ± 18.2	−2.4 (−6.1, 1.4)	0.217
TIR (70–140 mg/dl) (%)	37.0 ± 11.2	38.6 ± 12.5	−1.6 (−4.4, 1.2)	0.264	35.7 ± 10.4	37.5 ± 11.5	−1.8 (−4.5, 1.0)	0.202	41.1 ± 19.2	42.1 ± 19.9	−0.9 (−5.0, 3.1)	0.648
*Hyperglycemia*												
TAR (>140 mg/dl) (%)	52.7 ± 16.1	54.8 ± 15.8	−2.2 (−5.5, 1.2)	0.208	55.4 ± 15.1	57.3 ± 14.6	−1.9 (−5.3, 1.6)	0.285	44.4 ± 24.3	47.3 ± 23.0	−2.9 (−6.9, 1.1)	0.149
TAR (>180 mg/dl) (%)	30.9 ± 14.8	30.6 ± 13.6	0.3 (−2.8, 3.4)	0.832	32.6 ± 14.3	31.8 ± 13.1	0.9 (−2.3, 4.1)	0.585	25.7 ± 19.8	27.2 ± 18.7	−1.5 (−5.2, 2.3)	0.430
TAR (>250 mg/dl) (%)	7.4 (3.1,10.2)	6.9 (2.9, 14.2)	−0.7 (−2.1, 0.8)	0.412	8.0 (3.9, 11.7)	8.2 (3.4, 13.8)	−0.4 (−2.0, 1.3)	0.852	2.0 (0.0, 6.4)	2.3 (0.0, 13.0)	−1.9 (−3.3, −0.5)	0.015
HBGI	6.7 ± 3.3	7.0 ± 3.3	−0.3 (−1.0, 0.4)	0.388	7.1 ± 3.3	7.3 ± 3.1	−1.8 (−0.9, 0.6)	0.628	5.0 (1.6, 7.6)	4.6 (2.5, 9.2)	−0.7 (−1.5, 0.0)	0.059
*Glycemic variability*												
SD (mg/dl)	60.5 ± 11.5	59.7 ± 13.5	0.8 (−2.0, 3.6)	0.582	60.8 ± 10.6	59.5 ± 12.6	1.2 (−1.5, 4.0)	0.363	53.1 (39.3,61.8)	50.8 (42.4, 67.3)	−2.1 (−5.8, 1.7)	0.274
CV (%)	40.9 ± 9.3	38.7 ± 8.5	2.1 (0.4, 3.9)	0.017	39.9 ± 8.5	37.8 ± 8.1	2.1 (0.5, 3.7)	0.012	37.4 (32.4,45.9)	38.9 (28.7, 44.9)	1.11 (−1.7, 3.9)	0.432
MAGE (mg/dl)	153.9 ± 30.6	151.2 ± 33.8	2.7 (−4.6, 10.0)	0.459	—	—	—	—	—	—	—	—
MODD (mg/dl)	64.7 ± 16.8	60.8 ± 17.3	3.8 (−0.1, 7.8)	0.057	—	—	—	—	—	—	—	—

*Note*: The differences were calculated by subtracting FSL from iPro2 estimates. *p*‐values were calculated with paired‐samples T‐test and Wilcoxon's paired ranked test for normally distributed parameters and non‐normally distributed parameters.

Abbreviations: CI, confidence interval; CV, coefficient of variation; FSL, FreeStyle Libre; HBGI, high blood glucose index; LBGI, low blood glucose index; MAGE, mean amplitude of glycemic excursions; MODD, mean of daily differences; TAR, time above range; TBR, time below range; TIR, time in range.

## 4 DISCUSSION

Nowadays, multiple brands of CGM have been put into clinical use but the consensus provides the same uniform rules for all devices.^3^, ^19^ Therefore, whether the clinical performance of brands is similar is necessary to be discussed. In our study, two commercially available CGM systems indicated for nonadjunctive use were tested in parallel for 14 days to evaluate the accordance in metrics among adult patients with T1DM. The results showed that the agreement between the two devices was acceptable but there were still significant differences especially in the hypoglycemic and target range.

Metrics in conjunction with HbA1c for glycemic control assessment are of great importance.^23^ The TIR is one of the most meaningful indexes for clinical glycemic control and the assessment of the risk of diabetic complications because of its advantages in straightforward definitions and easy calculation methods.^24^, ^25^, ^26^, ^27^ In this study, even though accuracy of the two devices in the euglycemic range was acceptable and the correlation among the paired iPro2‐FSL data sets was high, the metrics calculated in the respective whole data sets were significantly different. The reason for this deviation might be the time interval of data collection. As reported in the previous studies, there was a physiological time delay for the glucose assessment because CGM systems measured glucose levels in the interstitial fluid in the subcutaneous adipose tissue instead of directly in the blood.^28^ Even under reproducible experimental conditions and independent of a specific CGM system, there was still great variability in the time delay between individual subjects. Therefore, as the time intervals were two times longer in the FSL group than that in iPro2, the smaller datasets collected in FSL might increase the random bias, which further reduced the accuracy and reliability for the related clinical metrics. Furthermore, with the time interval of data collection potentially influencing the clinical metrics, whether the large glycemic fluctuation influenced the accordance of clinical metrics between the two devices should be further discussed.

Time spent in the hypoglycemic range, including the TBR < 54 mg/dl and TBR < 70 mg/dl, were also vital metrics in clinical practice as reduction in hypoglycemia could help reduce acute damage, risk of death, and decline in cognitive function.^29^, ^30^, ^31^, ^32^ In this study, of note, regardless of the collection time period, the differences in hypoglycemic metrics between the two devices were all significant. In Table 3, the discordance of glucose values in the extracted pairs of data sets between iPro2 and FSL was also observed. Furthermore, different from the deduction in the TIR deviation, these discordances in hypoglycemic range in our study might be largely due to the accuracy of the data collection. In our study, as presented in Table 2, taking SMBG as reference, the median ARD in the hypoglycemic group was significantly lower for iPro2, implying the FSL might underperform in hypoglycemic range. Besides, the performance of CGM devices in the hypoglycemic range had also been criticized.^33^ In most published studies assessing the accuracy, the median ARD in the hypoglycemic range was usually exceeding the acceptable value of 20%. Therefore, with lower performance of the devices in the hypoglycemic range, the related clinical metrics could be less reliable, which indicated that the application of devices in clinical trials should consider the models especially those assessing the hypoglycemia. Furthermore, the next generation of the CGM systems should improve their performances in hypoglycemia.

Similar results discussing in the measurement accuracy between FSL‐pro and iPro2 had been reported by Kumagai et al whereas our study expanded the analysis on discrepancies in glycemic metrics,^34^ which is more straightforward and practical in clinical practice. This is also highlighted in the findings in our study, which could remind us of a further consideration on the model heterogeneity. Besides, when discussing the accuracy, the wider range of glucose levels reported in our study instead of concentrating mostly on hyperglycemia (>250 mg/dl) in Kumagai et al's work might be more rationale and reliable. There were also several limitations in our study. First, even though the point of care was used as the reference value in our study, but the testing numbers might be inadequate, especially in the hypoglycemic range. This might be less reliable for the performance assessment and clinical metrics calculation. Second, the highest threshold between the two devices was different with only 400 mg/dl in the iPro2 system and 500 mg/dl in FSL. The discordance in the glucose threshold might result in the underestimation of the clinical metrics in iPro2, such as the mean/median glucose levels and so on. Third, the two devices were worn on different arms, which might also result in different glucose values.^35^

In the 14‐day wearing of different CGM systems simultaneously, even both datasets highly correlated with the SMBG values, FSL, and iPro2 provided different estimations on TIR, CV, and hypoglycemia‐related parameters, which reminded us of a further consideration of the model heterogeneity when making clinical decisions and clinical trial designs.

## DISCLOSURE

The authors declare that they have no conflict of interest.

## Supporting information


**FIGURE S1** Parkes error grid analyses.
