# Supplementary material for: Discrepancies in glycemic metrics derived from different continuous glucose monitoring systems in adult patients with type 1 diabetes mellitus
Source: J Diabetes. 2022 Jul 21;14(7):476–84. doi: 10.1111/1753-0407.13296 (PMC9310046; doi:10.1111/1753-0407.13296)
Supplement: Supplementary file 1 — FIGURE S1 Parkes error grid analyses. [file JDB-14-476-s001.docx]

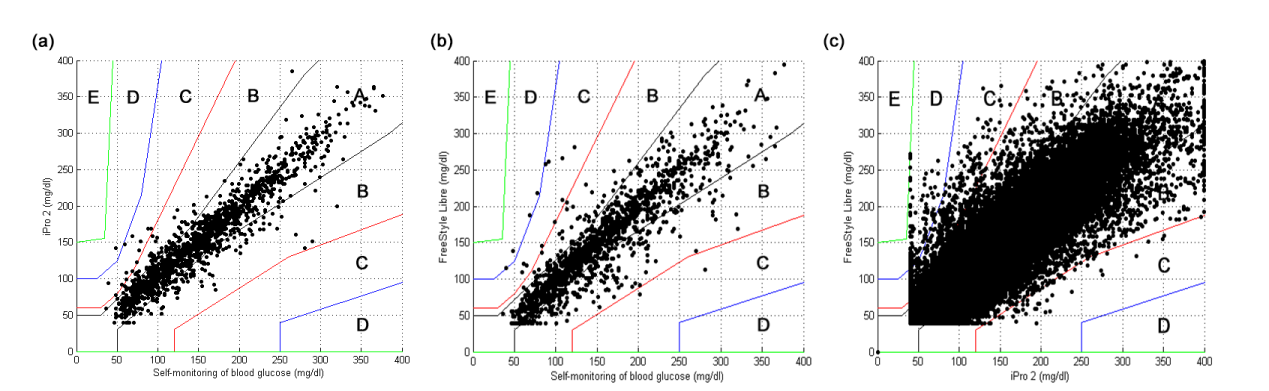


|  |  |  |  | Zones |  |  |  |
| --- | --- | --- | --- | --- | --- | --- | --- |
|  | A(%) | B(%) | A +B(%) | C(%) | D(%) | E(%) | Total |
| SMBG-iPro2 | 86.2  （n=1213） | 12.9  （n=182） | 99.1  （n=1395） | 0.9  （n=12） | 0.1  （n=1） | 0  （n=0） | 100  （n=1408） |
| SMBG-FSL | 80.5  （n=1133） | 18.4  （n=259） | 98.9  （n=1392） | 0.9  （n=12） | 0.3  （n=4） | 0  （n=0） | 100  （n=1408） |
| iPro2-FSL | 70.6  （n=28582） | 27.5  （n=11144） | 98.1  （n=39726） | 1.6  （n=649） | 0.3  （n=123） | 0  （n=0） | 100  （n=40498） |

**Fig. S1.** Parkes error grid analyses.

Parkes error grid analyses. (a) the SMBG and iPro2; (b) the SMBG and FSL; (c) iPro2 and FSL.

Abbreviations: SMBG, self monitoring of blood glucose; FSL, FreeStyle Libre.
